# Iterative Reweighted Noninteger Norm Regularizing SVM for Gene Expression Data Classification

**DOI:** 10.1155/2013/768404

**Published:** 2013-08-05

**Authors:** Jianwei Liu, Shuang Cheng Li, Xionglin Luo

**Affiliations:** ^1^Department of Automation, China University of Petroleum, Beijing 102249, China; ^2^Beijing Aerospace Propulsion Institute, Beijing 10076, China

## Abstract

Support vector machine is an effective classification and regression method that uses machine learning theory to maximize the predictive accuracy while avoiding overfitting of data. *L2* regularization has been commonly used. If the training dataset contains many noise variables, *L1* regularization SVM will provide a better performance. However, both *L1* and *L2* are not the optimal regularization method when handing a large number of redundant values and only a small amount of data points is useful for machine learning. We have therefore proposed an adaptive learning algorithm using the iterative reweighted *p*-norm regularization support vector machine for 0 < *p* ≤ 2. A simulated data set was created to evaluate the algorithm. It was shown that a *p* value of 0.8 was able to produce better feature selection rate with high accuracy. Four cancer data sets from public data banks were used also for the evaluation. All four evaluations show that the new adaptive algorithm was able to achieve the optimal prediction error using a *p* value less than *L1* norm. Moreover, we observe that the proposed *Lp* penalty is more robust to noise variables than the *L1* and *L2* penalties.

## 1. Introduction

Support vector machine (SVM) has been shown to be an effective classification and regression method that uses machine learning theory to maximize the predictive accuracy while avoiding overfitting of data [1]. *L2* regularization method is usually used in the standard SVM. It works well especially when the dataset does not contain too much noise. If the training data set contains many noise variables, *L1* regularization SVM will provide a better performance. Since the penalty functions are predetermined for data training, SVM algorithms sometimes work very well but other times are unsatisfactory. 

In many potential applications, the training data set also contains a large number of redundant values and only a small amount of data points is useful for machine learning. This is particularly more common in bioinformatics applications. 

In this paper, we propose a new algorithm for supervised classification using SVM. The algorithm uses an iterative reweighting framework to optimize the penalty function for which the norm is selected between 0 and 2, that is, 0 < *p* ≤ 2. We call it the iterative reweighted *p*-norm regularization support vector machine (IPWP-SVM). The proposed algorithm is simple to implement and has a fast convergence and improved stability. It has been applied to the diagnosis and prognosis of bladder cancer, lymphoma, melanoma, and colon cancer using publicly available data sets and evaluated by a cross-validation arrangement. The results from this proposed method provide more accurate functions than the rules obtained with classical methods such as the *L*1 and *L*2 norm SVM. The simulation results also reveal several interesting properties about the *p*-norm regularization behavior. 

The rest of this paper is organized as follows. The motivation of the variable selection of the *p*-norm will be formally introduced, followed by the IRWP-SVM algorithm development. Simulation results and results using real patient data sets will be discussed in Section 4. Finally, in Section 5 we provide a brief conclusion.

## 2. Motivation

Consider a training set of pairs *S* = {(*x*_*i*_,*y*_*i*_)}_*i*=1_^*m*^⊆*R* ^*n*^ × {±1} drawn independently identically and distributed (i.i.d.) from some unknown distribution *p*, where *x*_*i*_ ∈ *R* ^*n*^ is an *n*-dimensional input vector and *y*_*i*_ ∈ {−1, +1} is the corresponding target. Large-margin classifiers typically involve the optimization of the following function:

(1) $$\begin{array}{c} \arg\;\min f\left(X,Y,w\right)=\Phi \left(w\right)+a\cdot \Gamma \left(Y,w^{T}x_{i}\right), \end{array}$$

where Φ is a loss function, Γ is a penalty function, *X* = {*x*_1_^*T*^,…,*x*_*m*_^*T*^}^*T*^ ∈ *R* ^*m*×*n*^, *Y* = (*y*_1_,…,*y*_*m*_)^*T*^ ∈ {−1,+1}^*m*^, *w* = (*w*_1_^*T*^,…,*w*_*n*_^*T*^)^*T*^ ∈ *R* ^*n*^, and *a*  is a scalar. 

Equation (1) can be rewritten as

(2)   arg min⁡ Φ(w)subject  to  Γ(Y,wTxi)<λ,

where *λ* is a user-selected limit. Equations (1) and (2) are asymptotically equivalent. The standard SVM classifier can be considered as another approach to solve the following problem:

(3)    min⁡||w||22subject  to  yi·(wTxi+b)≥1 i=1,…,m,

where *b* is a bias term.

Oftentimes, the target value *y*_*i*_ is determined by only a few input elements in the input vector with a large dimension. In other words, the dimension of a sample data set is significantly larger than the number of key input features which are useful for identifying the target. The weight vector will be a sparse vector with many zeros. In this situation, the optimization problem in (1) and (2) should be searching for a sparse vector which still allows for accurate correlation between the target and inputs. A simple way of identifying the less sparse vectors is to count the number of nonzero elements of *w*_*i*_. In other words, the actual objective function being minimized is the *L*0-norm of *W*. Therefore, (3) should be replaced with the *L*0-norm of *w*_*i*_ as

(4)   min⁡||w||0subject  to  yi·(wTxi+b)≥1 i=1,…,m.

This optimization problem is known as the regularization SVM where the complexity of the model is related to the number of variables involved in the model. Amaldi and Kann show that the above problem is NP-hard [2]. In order to overcome this issue, several modifications have been proposed to relax the problem in machine learning and signal processing [3–5]. Instead of *L*0-norm, (4) is modified to the following convex optimization problem:

(5)   min⁡||w||1subject  to  yi·(wTxi+b)≥1 i=1,…,m.

It turns out that for linear constraints satisfying certain modest conditions, *L0*-norm minimization is equivalent to *L1*-norm minimization, which leads to a convex optimization problem for which there exist practical algorithms [6]. The presence of the *L1* term encourages small components of *x* to become exactly zero, thus promoting sparse solutions [7, 8].

**Figure 1:**
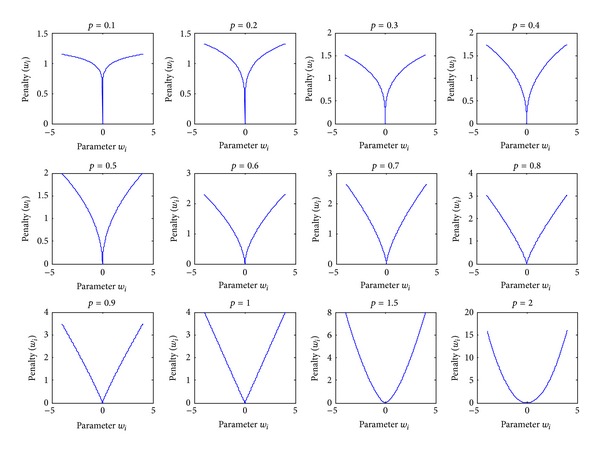
Illustration of the penalty functions Γ(*Y*, *w* ^*T*^ *x*_*i*_) = *a*_*i*_|*w*|^*p*^,  *a*_*i*_ = 1.

Another interesting possibility is to minimize the *Lp*-norm, where 0 < *p* < 1, which should yield sparser solutions than with *p* = 1 and *p* = 2. Such an optimization problem is nonconvex and likely has many local solutions, which make its use technically more challenging than that of the more common *L1* or *L2* norm. However, there may be an advantage in the case of data inconsistencies caused by noises. Despite the difficulties raised by the optimization problem, good empirical results were reported in signal reconstruction [9], SVM classification [10], and logistic regression [11].  Figure 1 provides an illustration of the following penalty functions:

(6) $$\begin{array}{c} \Gamma \left(Y,w^{T}x_{i}\right)=a_{i}^{}{\left|w\right|}_{}^{p} \end{array}$$

for *a*_*i*_ = 1 and *p* = {0.1,0.2,0.3,0.4,0.5,0.6,0.7, 0.8, 0.9,1.0,1.5,2.0}. When 0 < *p* < 1, *p*-norm is known as the bridge penalty function. This type of penalty has been used in signal processing fields [12, 13] and popularized further in statistical community [14, 15]. The special case of *p*-norm where 0 < *p* < 1 can be considered a quasi-smooth approximation of the *L0*-norm.

Meanwhile, several works have provided some theoretical guarantees on the use of the *Lp* penalty which justifies the use of such a penalty for variable selections [16–19]. Chartrand and Yin [20, 21] and Candés et al. [22] proposed some algorithms that were applied in the context of compressive sensing and share the same idea of solving a non-convex problem using an iterative reweighted scheme until complete convergence.

## 3. Iterative Reweighted *p*-Norm Regularization SVM 

In this section, we propose our iterative reweighted *p*-norm regularization algorithm. Given a set of datasets *X* = {*x*_1_^*T*^,…,*x*_*m*_^*T*^}^*T*^and their labels *Y* = (*y*_1_,…,*y*_*m*_)^*T*^ ∈ {−1,+1}^*m*^, the goal of the binary-class classification in SVM is to learn a model that assigns the correct label to the test samples. This can be thought of as a learning function = sign⁡(*w* ^*T*^ *x*_*i*_ + *b*): *X* → *Y* which maps each instance *x*_*i*_ ∈ *R* ^*n*^ to an estimated value ${\hat{y}}_{i}$. In this paper, for simplicity and brevity, only two classification problems will be shown. The data set is assumed to be linearly separable. Then, the problem of hard-margin, support vector machine using *p* norm regularization can be represented by the following optimization problem:

(7)   min⁡12||w||ppsubject  to  yi(wTxi−b)≥1, i=1,…,m,

where *w* ∈ *R* ^*n*^ and 0 < *p* ≤ 2. By rearranging the constraints in (7), the optimization becomes

(8)   min⁡12||w||ppsubject  to  yi([w−b]T[xi1])≥1   for  i=1,…,m.

Now define

(9) $$\begin{array}{c} w:=\left[\begin{array}{c} w\\ -b \end{array}\right],\;\;x_{i}^{}:=\left[\begin{array}{c} x_{i}\\ 1 \end{array}\right]\;\text{for}\;i=1,\ldots ,m. \end{array}$$

By substituting definition (9) into (8), we can rewrite the minimization in (8) as

(10)   min⁡12||w||ppsubject  to  yiwTxi≥1    for  i=1,…,m.

The Lagrangian function can be obtained as follows:

(11) $$\begin{array}{c} L\left(w,a\right)=\frac{1}{2}\sum_{j=1}^{n}{\left|w_{j}\right|}_{}^{p}-\sum_{i=1}^{m}a_{i}^{}y_{i}^{}w_{}^{T}x_{i}+\sum_{i=1}^{m}a_{i}. \end{array}$$

Therefore

(12) $$\begin{array}{c} L\left(w,a\right)=\frac{1}{2}\sum_{j=1}^{n}{\left|w_{j}\right|}_{}^{p-2}w_{j}^{2}-\sum_{i=1}^{m}a_{i}^{}y_{i}^{}w_{}^{T}x_{i}+\sum_{i=1}^{m}a_{i}. \end{array}$$

Define the following two variables:

(13) $$\begin{array}{c} V=\mathrm{diag}\left[{\left|w_{1}\right|}_{}^{p-2},\ldots ,{\left|w_{n}\right|}_{}^{p-2}\right],\\ I=\mathrm{diag}\left[\begin{array}{ccc} 1 & \cdots & 1 \end{array}\right]. \end{array}$$

Using the matrix and vector notation, we can rewrite (12) as

(14) $$\begin{array}{c} L\left(w,a\right)=w^{T}Vw-a^{T}\mathrm{diag}\left(Y\right)Xw+Ia. \end{array}$$

The corresponding dual is found by the differentiation with respect to the primal variable *w* and, that is,

(15) ∂L∂w=wTV−aTdiag⁡(Y)X=0⇒wT=aTdiag⁡(Y)XV−1.

Substituting *w* into the Lagrangian function, one may obtain

(16) LD(a)=12a·diag⁡(y)·XV−1VV−1XT·diag⁡(y)·a −a·diag⁡(y)·XV−1XT·diag⁡(y)·a+Ia=−12a·diag⁡(y)·XV−1XT·diag⁡(y)·a+Ia.

Therefore, the Wolfe dual problem becomes

(17) max⁡a{LD(a)} =max⁡a{−12aTdiag⁡(Y)XV−1XTdiag⁡(Y)a+Ia}.

The above optimization problem is a QP problem of variable *a* and it takes a form similar to the dual optimization problem for training support vector machines. The corresponding minimization problem becomes

(18) min⁡a{LD(a)} =min⁡a{12aTdiag⁡(Y)XV−1XTdiag⁡(Y)a−Ia}subject  to  ai≥0 for  i=1,…,m    ∑i=1maiyi=0 for  i=1,…,m.

Let *S* denote the set of indices of the support vector, where *a*_*i*_ ≠ 0; |*S*| is the cardinality of *S*. According to the Karush-Kuhn-Tucker (KKT) conditions,

(19) $$\begin{array}{c} a_{i}^{}\left[y_{i}^{}\left(w_{}^{T}x_{i}-b\right)-1\right]=0, \end{array}$$

where either *a*_*i*_ = 0 or *y*_*i*_(*w* ^*T*^ *x*_*i*_ − *b*) = 1 for *i* = 1,…, *m*. Therefore,

(20) $$\begin{array}{c} b=\frac{1}{\left|S\right|}\sum_{k\in S}\left(w_{}^{T}x_{k}^{}-y_{k}^{}\right). \end{array}$$

The final discriminant function is

(21) f(w,b)=sign⁡(wTxi+b)=sign⁡(aTdiag⁡(Y)XV−1XT−b).

### 3.1. Implementation of the IRWP-SVM

There exists a large body of literature on solving QP wolf dual problems represented by (18). Several commercial software programs are also available for QP optimization. However, these mathematical programming approaches and software are not suitable for SVM problems fortunately, and the iterative nature of the current SVM optimization problem allows us to derive tailored algorithms which result in faster convergence with small memory requirements even for problems with large dimensions. Currently, the following four types of implementation have been proposed.

*Iterative Chunking*. In 1982, Vapnik proposed an iterative chunking method, that is, working set method, making use of the sparsity and the KKT conditions. At every step, the chunking method solves the problem containing all nonzero *a*_*i*_ plus some of the *a*_*i*_ violating the KKT conditions.

*Decomposition Method*. The decomposition method has been designed to overcome the problem in which the full kernel matrix is not available. Each iteration of the decomposition method optimizes a subset of coefficients and leaves the remaining coefficients unchanged. Iterative chunking is a particular case of the decomposition method.

*Sequential Minimal Optimization*. The sequential minimal optimization algorithm proposed by Platt selects working sets using the maximum violating pair scheme, that is, always using two elements as working set size. 

*Coordinate Descent Method*. This method iteratively updates a block of variables. During each iteration, a nonempty subset is selected as a block and the corresponding optimization subproblem is solved. If the subproblem has a closed-form solution, it neither uses any mathematical programming package nor needs any matrix operations.

In our study, we have applied Platt's “sequential minimal optimization” learning procedures to solve the QP wolf dual problems in (18). Sequential minimal optimization is a fast and simple training method for support vector machines. The pseudocode is given in Algorithm 1. Specifically, given an initial point (*w* ^(0)^, *b* ^(0)^), the IRWP-SVM computes (*w* ^(*t*+1)^, *b* ^(*t*+1)^) from (*w* ^(*t*)^, *b* ^(*t*)^) by cycling through the training data and iteratively solving the problem in (18) for only two elements which are composed of the maximum violating pair at a time.

## 4. Experiments and Discussion

Both simulation data and clinical data have been used to illustrate the IRWP-SVM. In particular, the results to follow will show that the IRWP-SVM is able to remove irrelevant variables and identify relevant (sometimes correlated) variables when the dimension of the samples is typically larger than the number of training points.

### 4.1. IRWP-SVM for Feature Selection in Simulation

We start with an artificial problem which is taken from the work by Weston et al. [23]. We generated artificial data sets as in [23] and followed the same experimental protocol in the first experiment. All samples were drawn from a multivariate normal distribution: the probability of *y* = 1 or −1 was equal. One thousand samples with 100 features were generated. Six dimensions out of 100 were relevant. These features are composed of three basic classes.

- The first class features are relevant features.
- The second class features are irrelevant features.
- The remaining features are noise.

Supposing that a sample is defined as *x*_*i*_ = (*x*_*i*,1_, *x*_*i*,2_,…, *x*_*i*,100_), then the first class features were drawn as (*x*_*i*,1_, *x*_*i*,2_, *x*_*i*,3_) with a probability of 0.7 and (*x*_*i*,4_, *x*_*i*,5_, *x*_*i*,6_) with a probability of 0.3, the second class features were drawn as (*x*_*i*,1_, *x*_*i*,2_, *x*_*i*,3_) with a probability of 0.3 and (*x*_*i*,4_, *x*_*i*,5_, *x*_*i*,6_) with a probability of 0.7, and the remaining features were drawn as (*x*_*i*,7_, *x*_*i*,8_,…, *x*_*i*,100_) with a probability of 1. The first class three features were drawn successively as *y* · *N*(3,1) distribution, and *y* · *N*(2.2,1) distribution, *y* · *N*(1.4,1) distribution, and the second class three features were drawn as *N*(0,1) distribution. The remaining 94 features were drawn as *N*(0,20) distribution. The inputs are then scaled to have a mean of zero and a standard deviation of one.

We used IRWP-SVM for the feature selection. To find out how the prediction error rate and feature selection error rate can be affected by the different training and validation set sizes, we conducted three sets of experiments on the data sets with the following combinations:

- 250 training samples + 750 validation samples,
- 500 training samples + 500 validation samples,
- 750 training samples + 250 validation samples.

Tables 1(a) and 1(b) summarize the results using two different criteria: prediction error rate and feature selection error rate. All results reported in the tables are averages over at least 100 independent trials. One may expect that a smaller *p* should work best in a setting where the number of relevant features is very small.

When the training + validation set size is 250 + 750, the highest prediction error rate is 0.47%, the lowest prediction error rate is 0.38%, the highest feature selection error is 8.33%, and the lowest feature selection error is 2%. When the training + validation set size is 500 + 500, the highest prediction error rate is 0.37%, the lowest prediction error rate is 0.28%, the highest feature selection error is 2.67%, and the lowest feature selection error is 0. For *p* = 0.7, 0.8, 0.9, and 1.0, the feature selection error is 0%. When the training + validation set size is 750 + 250, the highest prediction error rate is 0.37%, the lowest prediction error rate is 0.26%, the highest feature selection error is 0.50%, and the lowest feature selection error is 0. For *p* = 0.7, 0.8, 0.9, and 1.0, the feature selection error is 0%. The prediction accuracy rate is in between 99.5% and 99.8%. For *p* = 0.4, 0.5, 0.6, 0.8, 0.9, and 1.0, the feature selection error is 0%. One can see that the feature selection error is sensitive to changes in the *p* value. When 0 < *p* < 1, *p*-norm regularization SVM is a sparse model, and the feature selection error is sensitive enough to select the specified *p*-norm SVM model for improving the prediction accuracy.

**Figure 2:**
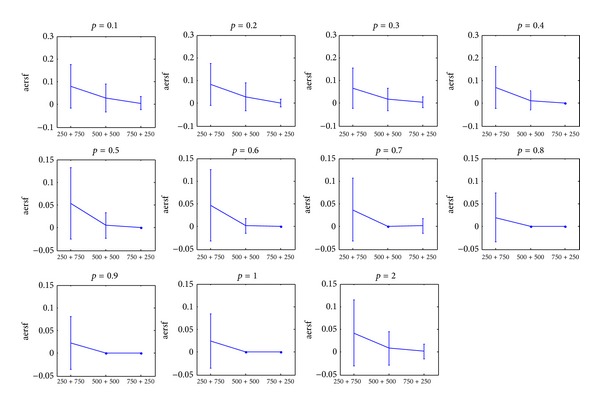
The average error rate of the selected features for different training + validation data sets at different *p*-norm values. (“aersf” represents the average error rate of selected features).

Figure 2 represents the error rate of feature selection for different *p*-norm values. Each subfigure consists of three data points which represent, respectively, the feature selection error rate when training + validation set size is 250 + 750,  500 + 500, and 750 + 250. The error bar is 2 times the standard deviation. With the increasing ratio of training and validation set sizes, the average value of feature selection error rate first decreased and then became stable. When the ratio of training and validation set size reached 500 + 500, the feature selection error rate reached its lowest point. To sum up, the sensitivity of the feature selection error rate of IRWP-SVM algorithm decreases when more training samples are used.

**Figure 3:**
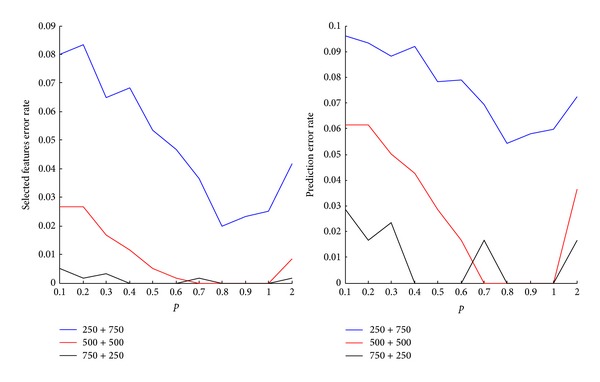
The trend graph of error rate for feature selection and prediction error rate on artificial datasets.

Figure 3 shows another perspective of the error rate trends. In summary, considering both the error rate for feature selection and the prediction error rate, *p* = 0.8 appears to be more suitable for the data. Our IRWP-SVM algorithm is highly accurate and stable, is able to remove irrelevant variables, and provides robustness in the presence of noises.

### 4.2. IRWP-SVM for Four Clinical Cancer Datasets

A major weakness of the *L2*-norm in SVM is that it only predicts a cancer class label but does not automatically select relevant genes for the classification. In this section, four experiments on four real cancer datasets were used to demonstrate the *p*-norm regularization support vector machine which can automatically select the *p* value and identify the relevant genes for the classification.

Table 2 shows the information of the four real cancer datasets and training + validation set size used in our evaluation. The bladder cancer dataset consists of 42 training and 15 validation data sets (http://www.ihes.fr/~zinovyev/princmanif2006/), a total of 57 sample sets. The dimension of each sample vector is 2215. The melanoma cancer dataset consists of 58 training and 20 validation data sets (http://www.cancerinstitute.org.au/cancer_inst/nswog/groups/melanoma1.html), a total of 78 sample sets. The dimension of each sample vector is 3750. The lymphoma cancer dataset consists of 72 training and 24 validation data (http://llmpp.nih.gov/lymphoma/data/rawdata/), a total of 96 samples. The dimension of each sample vector is 4026. The colon cancer dataset consists of 46 training and 16 validation data (http://perso.telecom-paristech.fr/~gfort/GLM/Programs.html), a total of 62 samples. The dimension of each sample vector is 2000.

Table 3 is the prediction error rate and selected feature number for *p* = 0.25, 0.5, 0.75, 1.0, and 2.0. All results reported here are averages over at least 100 independent trials.

#### 4.2.1. Bladder Dataset

**Figure 4:**
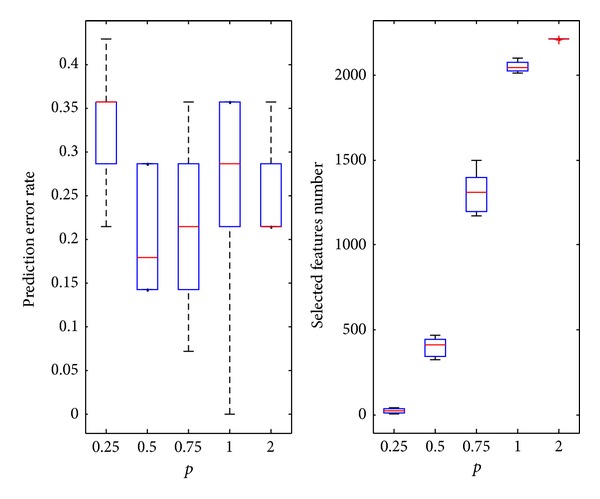
The experimental results on bladder datasets.

In Figure 4, *p* = 0.5 resulted in the minimum prediction error rate. As the value increases, the number of features gradually increases. The upper limit is the maximum number of features of the original data: 2215. For *p* = 0.5, the average number of the features is 403.2, and the average prediction error rate is 20% which is also the value of the optimal point.

#### 4.2.2. Melanoma Dataset

**Figure 5:**
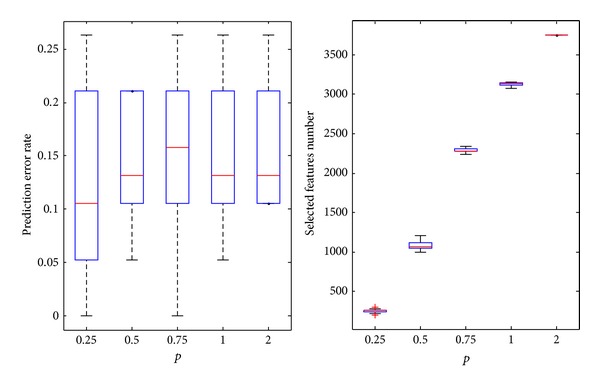
The experimental results on melanoma datasets.

In Figure 5, the predicted error rate at first increases and then decreases. The value *p* = 0.25 provides the minimum average error rate. As *p* increases, the number of features gradually increases (the upper limit is the maximum number of features in the original data, i.e., 3750). The average number for the selected features is 256.5 for *p* = 0.25. The average prediction error rate is 12.11%. It is also the value of the optimal point. The selected features are only 6.84% of the number of total features.

#### 4.2.3. Lymphoma Dataset

**Figure 6:**
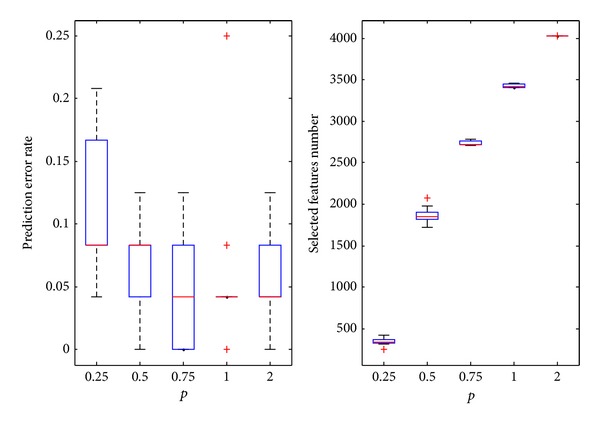
The experimental results on lymphoma datasets.

In Figure 6, the predicted error rate at first decreases and then increases. *p* = 0.75 provides the minimum average error rate. The upper limit is the maximum number of features in the original data set that is, 4026. For *p* = 0.75, the average number for the selected features is 2734.6, and the average prediction error rate is only 5% at the optimal *p* value. The average predicted error rate is 5.83% at *p* = 1, slightly higher than 5%. The data set also has a number of outliers. The stability of the predicted error at *p* = 1 is less than that of *p* = 0.75. The average number for the selected features is 3426.3, significantly higher than 2734.6. Therefore, the IRWP-SVM algorithm that selected *p* = 0.75 as the *p*-norm regularization is better than the *L*1 norm SVM.

#### 4.2.4. Colon Dataset

**Figure 7:**
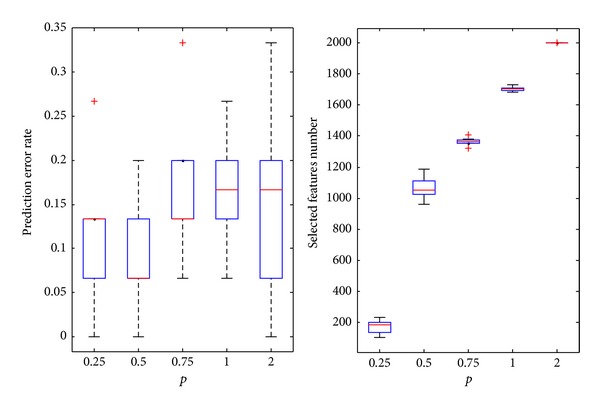
The experimental results on colon datasets.

In Figure 7, as *p* increases, the predicted error rate at first decreases and then increases. The average error rate achieved a minimum at *p* = 0.5. As *p* increases, the number of features gradually increases. The upper limit is the maximum number of features in the original data of 2000. For *p* = 0.5, the average number for the selected features is 1067.5, and it does not have any outlier. The selected features are only 53.4% of the number of total features, and thus the prediction time is significantly reduced.

#### 4.2.5. Comparison

 In this section, we compare the *L0*-norm regularized SVM (*L*0-SVM), the *L1*-norm regularized SVM (*L1*-SVM), the *L2*-norm regularized SVM (*L2*-SVM), random forest and the IRWP-SVM. We use random forest in WEKA 3.5.6 software developed by the University of Waikato in our experimental comparison. Each experiment is repeated 60 times. For *L0*-SVM, *L1*-SVM, and *L2*-SVM, the tuning parameters are chosen according to 10-fold cross validation, and then the final model is fitted to all the training data and evaluated by the validation data. The feature selection error is the minimum error when choosing the subsets of different sizes of genes. The means of the prediction error and feature selection error are summarized in Table 4. As one can see in the table, the IRWP-SVM seems to have the best prediction performance.

## 5. Conclusions 

We have presented an adaptive learning algorithm using iterative reweighted *p*-norm regularization support vector machine for 0 < *p* ≤ 2. The proposed *Lp* regularization algorithm has been shown to be effective and able to significantly improve the classification performance on simulated and clinical data sets. Four cancer data sets were used for the evaluation. Based on the clinical data sets, we have found the following.

- The IRWP-SVM is a sparse model; the smaller the *p* values, the more sparse the model.
- The experiments show that the prediction error of the IRWP-SVM algorithm is small and the algorithm is robust.
- Different data require different *p* value for optimization. The IRWP-SVM algorithm can automatically select the value in order to achieve high accuracy and robustness.

The IRWP-SVM algorithm can be easily used to construct arbitrary *p*-norm regularization SVM algorithm (0 < *p* ≤ 2). It can be used as a classifier for many different types of applications.

## Figures and Tables

**Algorithm 1:**
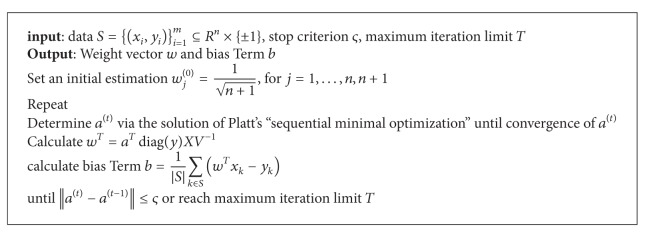
Iterative re-weighted *p*-norm regularization SVM.

**Table:** (a)

Training + validation	0.1	0.2	0.3	0.4	0.5
250 + 750					
Pe	0.47	0.43	0.45	0.42	0.38
Fse	8.00	8.33	6.5	6.83	5.33
500 + 500					
Pe	0.37	0.34	0.32	0.31	0.29
Fse	2.67	2.67	1.67	1.17	0.50
750 + 250					
Pe	0.35	0.30	0.32	0.34	0.31
Fse	0.50	0.17	0.33	0	0

**Table:** (b)

Training + validation	0.6	0.7	0.8	0.9	1.0	2.0
250 + 750						
Pe	0.38	0.38	0.41	0.41	0.40	0.42
Fse	4.67	3.67	2.00	2.33	2.50	4.17
500 + 500						
Pe	0.28	0.33	0.28	0.30	0.31	0.34
Fse	0.17	0	0	0	0	0.83
750 + 250						
Pe	0.31	0.28	0.26	0.28	0.37	0.34
Fse	0	0.17	0	0	0	0.17

**Table 2:** Four clinical cancer datasets and training + validation sizes.

Dataset	Training + validation sample set sizes	Dimension in each vector sample
Bladder	42 + 15	2215
Melanoma	58 + 20	3750
Lymphoma	72 + 24	4026
Colon	46 + 16	2000

**Table 3:** The prediction error rate (Pe%) and selected feature number (Sfn) for different *p* values.

Datasets	(%)	*p* = 0.25	*p* = 0.50	*p* = 0.75	*p* = 1.00	*p* = 2.00
Bladder	Pe	32.86	20.00	20.71	26.43	25.00
	Sfn	24.70	403.2	1312.5	2051.6	2211.9
Melanoma	Pe	12.11	13.68	14.74	14.74	15.26
	Sfn	256.5	1083.4	2290.5	3125.5	3750
Lymphoma	Pe	11.25	6.67	5.00	5.83	6.25
	Sfn	347.5	1870.9	2734.6	3426.3	4025.2
Colon	Pe	12.67	10.00	16.00	16.00	14.67
	Sfn	174.7	1067.5	1364.7	1702.1	1999.9

**Table 4:** Comparison of the prediction error (Pe%) and feature selection error (Fse%) on all four clinical cancer datasets.

Datasets	IRWP-SVM	L2-SVM	L1-SVM	L0-SVM	Random forest
Bladder					
Pe	20.1	23.6	26.41	28.41	22.56
Fse	3.47	33	4.21	9.28	—
Melanoma					
Pe	13.68	14.71	14.11	18.13	11.13
Fse	0.25	23.8	1.5	6.34	—
Lymphoma					
Pe	6.67	7.39	6.10	10.10	3.28
Fse	1. 1	14.3	1.62	5.91	—
Colon					
Pe	10	12.2	16.39	11.18	19.75
Fse	2.34	14.7	3.10	8.21	—
